# Clinical Medicine in Louisville

**Published:** 1849-05

**Authors:** 


### THE WESTERN JOURNAL.

THIRD SERIES.—VOL. III.—NO. V.

LOUISVILLE, MAY 1, 1849.

### CLINICAL MEDICINE IN LOUISVILLE.

An ordinance was passed by the city council of Louisville, on the 26th of April, establishing the proper relations between the Marine Hospital and the medical department of the University, providing for the erection of a spacious theater in connection with the hospital, and the appointment of a lecturer, whose business it will be to give daily attendance to the sick, and clinical instruction to medical students. This subject, which has been agitated so long, and been found so difficult to settle satisfactorily, is now, we trust, placed upon a secure basis, and one which will prove beneficial to the hospital and the medical school. With a lecturer of talents, learning, and tact as a teacher, we do not see why clinical instruction in our hospital may not be rendered as profitable as it can anywhere be made to a large class. An ample number and variety of medical cases is furnished by the wards, and the late ordinance provides for the erection of a lecture-room spacious enough to seat five hundred students. The completion of this arrangement is a matter of congratulation with the friends of the school. It places the institution upon ground where alone a medical school, at the present day, can hope to command enduring success. It secures to it the means of imparting to its pupils instruction in clinical medicine.

